# Cardioprotection for radiation-induced heart disease in breast cancer patients

**DOI:** 10.3389/fphar.2025.1742769

**Published:** 2026-01-06

**Authors:** Sabina Mędrek, Kasper Rolek, Jakub Homotnik, Sebastian Szmit

**Affiliations:** 1 Department of Cardio-Oncology, Centre of Postgraduate Medical Education, Warsaw, Poland; 2 Department of Cardiology and Cardio-Oncology Unit, Subcarpathian Oncological Center, Brzozow, Poland; 3 Cardio-Oncology Unit, Maria Skłodowska-Curie National Research Institute of Oncology, Warsaw, Poland

**Keywords:** cardio-oncology, cardioprotection, coronary artery disease, heart disease, radiotherapy

## Abstract

Radiotherapy is an integral part of early breast cancer treatment, when breast-conserving surgery is performed. Heart disease caused by radiotherapy can include pericardial lesions, cardiac dysfunction, valvular defects, coronary artery damage, and cardiac arrhythmias. Risk factors primarily include those related to the mean heart dose of radiation, but with the development of modern radiotherapy techniques, this problem is optimistically decreasing. However, concurrent pharmacological anticancer therapies still significantly impact the vessels and heart. The key problem, however, is pre-existing heart disease, often based on atherosclerosis or arterial hypertension. Efforts should be made to ensure that radiotherapy produces at most permissive cardiotoxicity, which will not affect the quality of life or survival. Hence, effective pharmacotherapy for primary and secondary cardioprotection is searching.

## 1 Introduction

Breast cancer (BC) is the most common female malignancy worldwide (Siegel et al., 2024). According to the latest data from the National Cancer Registry (NCR), 21,554 new cases were recorded in 2022 in Poland, accounting for 23.6% of all malignancies diagnosed in women (Strona, 2025). Carcinoma of the male breast is rare, accounting for less than 1% of all cancer cases in men; in Poland, it corresponds to an annual estimate of about 140 cases (Strona, 2025; Fox et al., 2022). BC is also the second leading cause of cancer-related death among women, following lung cancer (LC). In 2022, 6,611 deaths were recorded in Poland, representing 14.9% of all cancer-related deaths among women (Strona, 2025). The aetiology of BC remains largely unclear. The most significant risk factors include female sex, advanced age, a positive family history of BC, and the presence of pathogenic gene variants, *BRCA1* and *BRCA2* mutations, as well as early menarche and late menopause. Modifiable risk factors include postmenopausal overweight and obesity, prolonged use of hormone replacement therapy, and long-term hormonal contraception (Brody et al., 2007). Individual cancer risk can be estimated using validated predictive models (Stark et al., 2019). The Gail model is the most widely used breast cancer risk assessment tool (Stevanato et al., 2022). This scale incorporates several clinical and demographic parameters, including current age, age at menarche, age at first live birth, first-degree family history of BC, number of previous breast biopsies, and atypical hyperplasia in biopsy. Approximately half of all cases occur in women with no identifiable risk factors other than female gender and age over 40 years (Who, 2025). This highlights the extremely complex interplay between environmental, lifestyle and genetic factors, as well as underscores the need for further research into the mechanisms underlying this disease.

### 1.1 Radiation therapy

Radiation therapy (RT) remains an essential component of BC management. It is most used in conjunction with systemic treatment and surgical intervention in cases of early-stage disease, as well as in the management of local recurrences. RT plays a pivotal role in adjuvant therapy following breast-conserving surgery. This combined treatment is no less effective than mastectomy and is currently the preferred treatment option (Veronesi et al., 2002; Moran et al., 2014). Postoperative RT reduces the risk of local recurrence and improves survival (Early Breast Cancer Trialists' Collaborative Group EBCTCG et al., 2011). Most commonly, beams with energies of 4–6 meV are used at a dose of 40 Gy in 15 fractions over 3 weeks (Smith et al., 2011) or at 26 Gy in 5 fractions over 1 week (Murray Brunt et al., 2020). Additionally, higher targeted doses may be administered to the tumour bed using photon beams, electron beams, or brachytherapy to reduce the risk of local recurrence (Bartelink et al., 2015).

### 1.2 Cardiovascular mortality among BC patients

Improvements in early diagnosis and advances in BC treatment have contributed to a reduction in the risk of cancer-related death in these patients (Sung et al., 2021). Therefore, it can be concluded that an increasing number of BC patients will die from conditions other than breast cancer. Cardiovascular disease (CVD) is the most common cause of death globally (Roth et al., 2020). A large cohort study has shown that for women aged ≥66 years who survived at least 5 years after breast cancer diagnosis, CVD became the leading cause of death by the 10-year mark, when the cumulative incidence of each was 5% (A et al., 2017). Another large cohort study found that CVD (15.9% (95% CI 15.6–16.2)), followed by BC (15.1% (95% CI 14.8–15.4)) was the leading cause of mortality across the entire study population of BC patients (Patnaik et al., 2011). Analysis of SEER data including 655,552 breast cancer cases has shown that the risk of CVD was significantly higher in BC patients compared to the general population and occurred mainly within 10 years of BC diagnosis (Luo et al., 2025). Multivariate analysis showed that RT was associated with an increased risk of CV death (RR ≈ 1.29 [95% CI: 1.13–1.48]) compared to women who did not receive such treatment (Cheng et al., 2017). Additionally, left‐sided RT was associated with a significantly increased risk of cardiac death compared with right‐sided treatment (RR 1.22, 95% CI 1.08–1.37, p = 0.002) (Cheng et al., 2017).

## 2 Radiation-induced heart disease

Thoracic radiotherapy, while significantly improving cancer survival, can cause radiation-induced heart disease (RIHD). The term encompasses pericardial disease, cancer treatment-related heart dysfunction (CTRCD), coronary artery disease, valvular heart disease, and conduction abnormalities (Ellahham et al., 2022). Furthermore, RIHD is exacerbated by systemic therapy and CV risk factors, including diabetes mellitus (DM), hypertension (HT), obesity, and dyslipidaemia. The estimated incidence of RIHD at 5–10 years post-treatment was 10%–30% and varied depending on RT duration and the type of cancer (Wang et al., 2019). The incidence of specific heart diseases in BC patients ranges from 0.5% to 37% (Wang et al., 2019).

Radiation-induced heart disease (RIHD) is a multifactorial process driven by a complex interplay of vascular, inflammatory, oxidative, and fibrotic mechanisms (Wang et al., 2019; Taunk et al., 2015). A central initiating event is endothelial injury, which occurs shortly after radiation exposure and leads to microvascular dysfunction, increased vascular permeability, pro-thrombotic changes, and chronic inflammation (Taunk et al., 2015; Madan et al., 2015). Ionizing radiation induces excessive production of reactive oxygen species (ROS), resulting in persistent oxidative stress, mitochondrial dysfunction, lipid peroxidation, and DNA damage in cardiomyocytes and vascular cells (Ping et al., 2020; Wang et al., 2020). These processes activate multiple pro-inflammatory and pro-fibrotic signaling pathways, including NF-KB and TGF-β/Smad, promoting fibroblast activation and excessive extracellular matrix deposition (Wang et al., 2020). Progressive myocardial fibrosis, together with apoptosis of cardiomyocytes and remodeling of coronary micro- and macrovessels, ultimately leads to diastolic and systolic dysfunction, ischemic heart disease, and heart failure (Šteiner, 2020). Importantly, these pathological processes may evolve silently over years or decades following radiotherapy, explaining the delayed clinical presentation of RIHD in long-term cancer survivors (Wang et al., 2019).

### 2.1 Pericardial diseases

Pericardial diseases are one of the most common and earliest manifestations of RIHD (Cuomo et al., 2016). RT can give rise to pericardial involvement by generating reactive oxygen species (ROS), which cause oxidative damage to pericardial cells, leading to increased membrane permeability, neutrophilic infiltration, and subsequent effusion (Pedersen et al., 2023). Cancer patients are diagnosed with pericardial conditions such as pericardial effusion, acute pericarditis, and constrictive pericarditis. Pericardial effusion, defined as the accumulation of fluid within the pericardial cavity, may result from direct tumour invasion or as a complication of anticancer therapies, including RT, chemotherapy (ChT), and immunotherapy (IT). It is one of the most common incidental findings in cancer patients, particularly in those with BC, and its presence has been linked with increased mortality (Chahine et al., 2021). Acute pericarditis may develop secondary to direct neoplastic infiltration or as a result of cancer treatments, IT or RT in particular. Management typically includes nonsteroidal anti-inflammatory drugs (NSAIDs), colchicine, and, in selected cases, corticosteroids or anti–interleukin-1 (IL-1) agents. In refractory or recurrent cases, pericardiocentesis, prolonged pericardial drainage, or pericardial window may be necessary. Constrictive pericarditis, although rare, most often develops as a late complication of RT. Its diagnosis is challenging and requires multimodal imaging, whereas surgical pericardiectomy remains the definitive treatment option.

### 2.2 Cardiac dysfunction

Historically, various terms and definitions have been used to describe the spectrum of cardiovascular toxicities associated with cancer therapy, leading to inconsistencies in diagnostic criteria and clinical management. The 2022 ESC Cardio-Oncology Guidelines established a unified definition of cancer therapy–related cardiac dysfunction (CTRCD), encompassing a wide range of clinical manifestations and an aetiological relationship with a wide range of cancer treatments, including ChT, targeted therapy, IT, and RT. CTRCD has been further classified as symptomatic and asymptomatic. Symptomatic CTRCD corresponds to clinically overt heart failure (HF), a syndrome defined by the presence of major symptoms such as dyspnoea and peripheral oedema, often accompanied by additional signs including elevated jugular venous pressure and pulmonary crackles. The asymptomatic form of CTRCD is typically classified based on left ventricular ejection fraction (LVEF): LVEF ≤40% (HF with reduced ejection fraction, HFrEF); LVEF 41%–49% (HF with mildly reduced ejection fraction, HFmrEF); and LVEF ≥50% (HF with preserved ejection fraction, HFpEF); a new relative reduction in global longitudinal strain (GLS) > 15% compared with baseline, ccTnI/cTnT>99 percentile, BNP ≥35 pg/mL, NT-proBNP ≥125 pg/mL, or new significant rise from baseline beyond the biological and analytical variation of the assay used (Lyon et al., 2022). Cardiac troponins and natriuretic peptides represent key biomarkers in the early detection and monitoring of CTRCD; their use enables prompt intervention before irreversible damage occurs (Tamburrini et al., 2025). Emerging biomarkers, such as microRNAs and soluble ST2, have shown potential as indicators of the risk of myocardial dysfunction in patients undergoing anticancer therapy. They hold promise for better risk prediction, but currently available evidence is insufficient for their routine use (Madan et al., 2015). The estimated prevalence of radiation-induced cardiomyopathy is >10%, with an increased incidence of HF compared to the general population (Belzile-Dugas and Eisenberg, 2021; Koutroumpakis et al., 2020). In a population-based case-control study published in 2017 involving women who developed HF following RT for breast cancer, 64% of cases presented with HFpEF (LVEF >50%), and 89% had an LVEF >40%, indicating a predominance of preserved or mildly reduced systolic function in this population (Saiki et al., 2017). This is likely due to damage to rapidly proliferating endothelial cells and inflammation during RT, which leads to impaired microcirculation, oxidative stress, myocarditis, and ultimately fibrosis. Cardiac dysfunction and progression of HF may also arise from coronary endotheliitis caused by comorbidities (Paulus and Tschöpe, 2013; Mohammed et al., 2015). Cardiomyocytes are nonproliferating cells, therefore they are resistant to radiation. Their damage secondary to infarction or other factors is the main mechanism of HFrEF (Bloom et al., 2016). Clinical symptoms of RT-induced cardiac fibrosis appear late, after approximately 10 years (Belzile-Dugas and Eisenberg, 2021; Finch et al., 2014). It therefore seems important to use other diagnostic modalities to allow for earlier detection of myocardial damage and possible implementation of cardioprotective treatment. The ESC Cardio-Oncology Guidelines further incorporate the assessment of the left ventricular global longitudinal strain and cardiac biomarkers in the definition of CTRCD. A reduction in ejection fraction and GLS was observed in left-sided BC patients with no prior history of cardiac treatment 6 months after RT (Ellahham et al., 2022). An attempt was also made to develop a predictive model for radiotherapy-induced cardiotoxicity, incorporating dosimetric, demographic, imaging, and clinical parameters (Talebi et al., 2025). The link between RT and changes in biomarker levels remains unclear; therefore, no specific recommendations have been offered for the prevention of radiation-induced heart damage (Pudil et al., 2020). In different studies, multivariate QRS analysis showed no association between mean heart dose [median (IQR): 2.87 Gy (2.05–3.94)] and post-RT biomarker levels (hsCRP, NT-proBNP, hsTnI) in left-sided BC patients treated with modern RT techniques (FIELD-W-FIELD IMRT/VMAT ± Deep Inspiration Breath Hold [DIBH]) (Chufal et al., 2024). In BC survivors with advanced HF refractory to pharmacotherapy, heart transplantation may be considered after a cancer remission period of 2–5 years (López-Fernández et al., 2024). Despite the higher incidence of secondary malignancies and infections in this population, survival rates are comparable to those of patients undergoing heart transplantation for other cardiomyopathies (López-Fernández et al., 2024).

### 2.3 Coronary artery disease

Advances in modern radiation therapy planning and the adoption of novel techniques can substantially reduce cardiac radiation exposure; however, even low cardiac exposure during breast cancer RT increase the risk of coronary events (Darby et al., 2013). The incidence of coronary artery disease (CAD) increases proportionally with the mean heart radiation dose. It has its onset a few years after exposure, and persists for at least 20 years (Darby et al., 2013), affecting up to 85% of patients (Chang et al., 2017a). Women with preexisting cardiac risk factors are at a greater absolute risk from RT than other women (Darby et al., 2013). Radiation-induced vascular injury is believed to promote atherosclerosis, particularly at high radiation doses (Chang et al., 2017b) and in experimental models (Wu et al., 2023). On the other hand, low and moderate doses may attenuate the inflammatory response and, consequently, reduce atherosclerotic plaque formation. Cardiac radiation at 5 Gy has been shown to exert beneficial effects on cardiac remodelling in both mice and in humans with HF (Pedersen et al., 2023). A Swedish cohort treated for BC between 1970 and 2003 showed a direct link between radiation dose and the location of coronary artery stenosis, the proximal right coronary artery (prox RCA), the mid and distal left anterior descending artery, and the distal diagonal branch (mdLAD + dD) in particular (Nilsson et al., 2012). Similar findings have been reported in other clinical trials (Wennstig et al., 2019; Jacob et al., 2019; Piroth et al., 2019). Patients with CV risk factors and a mean cardiac dose >5 Gy are at increased risk of cardiotoxicity and should be referred for Cardio-Oncology assessment. Coronary artery calcium (CAC) scores obtained from CT prior to planned radiotherapy may represent a valuable screening tool, warranting further investigation (Otero-Pla et al., 2025). The outcomes of percutaneous coronary intervention (PCI) for CAD may be comparable between patients with and without prior RT (Mitchell et al., 2021). Performing coronary artery bypass graft (CABG) may be challenging due to potential subclavian artery stenosis or LIMA atresia, which can limit the availability of this procedure (Zhang et al., 2019). In cases involving a heavily calcified (‘porcelain’) aorta, PCI is often the preferred treatment option (Zhang et al., 2019).

### 2.4 Valvular heart disease (VHD)

Radiation-induced VHD (RIVHD) is observed in nearly 81% of patients treated with thoracic RT (Zafar et al., 2020). The incidence and severity of VHD depend on multiple factors, primarily the radiation dose received. These patients have a 9.2-fold increased risk of requiring valvular surgery compared to non-irradiated individuals (Belzile-Dugas and Eisenberg, 2021). The overall incidence in BC patients ranges from 0.5% to 4.2% (Otero-Pla et al., 2025). The primary mechanism underlying valvular damage involves fibroblast activation and the release of fibroblast growth factor, resulting in fibrosis with or without calcification (Gujral et al., 2016). This can cause thickening and/or calcification of the valves, annulus, and supra- or subvalvular structures, giving rise to valvular stenosis and/or regurgitation. Left-sided valves are most commonly affected, regardless of dose distribution, suggesting that higher systemic pressure further contributes to valvular damage. Aortic regurgitation is the most common defect, followed by aortic stenosis. Since the aortic valve is typically closest to the radiation field, it is most frequently affected by both regurgitation and stenosis. Mitral and tricuspid valve abnormalities have also been reported. As opposed to rheumatic heart disease, mitral valve commissures and leaflet tips are typically spared with radiation (Hering et al., 2003). As the disease progresses, patients may require interventional or surgical valve replacement. The standardised incidence rate ratio for valve surgery compared with a healthy age and sex-matched population was 9.2 (95% confidence interval 8.1–10.3) (Gujral et al., 2016). However, the rates of mortality and adverse events were higher after both interventional and surgical valve replacement in RIVHD patients vs. controls (Belzile-Dugas and Eisenberg, 2021). Patients with prior thoracic RT and severe aortic stenosis have poorer outcomes after any type of valve replacement than those without a history of RT. Retrospective cohorts of these patients (Zhang et al., 2019; Donnellan et al., 2017) seem to have better outcomes after TAVR vs. surgical aortic valve replacement (SAVR), although a subgroup of patients at low surgical risk may potentially be successfully treated with SAVR. SAVR may be considered in patients at lower surgical risk (e.g., younger age), especially those with technical/anatomical challenges associated with TAVR or those who require additional surgical interventions such as pericardiectomy, multiple valve surgery, or CABG (Zhang et al., 2019).

### 2.5 Arrhythmias

The incidence of cardiac arrhythmias in patients undergoing RT ranges from 2% to 19% (Bedi et al., 2023). There are diverse, largely dose-dependent mechanisms leading to different types of cardiac arrhythmias. However, the observed relationship between the mean heart dose (MHD) and coronary complications cannot be directly extrapolated to arrhythmia. Few studies have assessed the risk of arrhythmia in relation to the mean cardiac exposure, cardiac substructure doses in particular. A study in LC patients treated with RT found that arrhythmic events showed significant borderline correlations between the mean whole heart dose and the right atrium dose, but not with the left ventricular or left atrial dose (Wang et al., 2017). Other studies demonstrated that the radiation dose to the right atrium may be a more significant parameter than the mean whole-heart dose in evaluating the risk of arrhythmia, and that right-sided irradiation is associated with greater exposure of the right atrium compared to other cardiac structures (Errahmani et al., 2022). Both the sinoatrial node (SAN) and the atrioventricular node (AVN) may be substantially exposed during breast VMAT, particularly in right-sided cases (Loap et al., 2023). Studies evaluating nodal exposure may help establish dose constraints and criteria for additional cardiac-sparing techniques, such as respiratory techniques or proton therapy, which could bring benefit in patients with underlying rhythmic or conduction disorders (Loap et al., 2023). It was also observed that, despite the moderate dose range received by the SAN and AVN in BC patients treated with 3D-CRT, SAN was the most exposed cardiac structure in right-sided BC. This finding may explain previous results indicating a higher risk of arrhythmia and conduction disturbances in right-sided vs. left-sided BC, as well as a potential association with the right atrial dose (Errah et al., 2022). A large registry-based cohort study involving 8,015 BC patients diagnosed between 2001 and 2008 in the Stockholm-Gotland region, and followed until 2017, demonstrated an increased long-term risk of arrhythmia and heart failure following BC diagnosis. The hazard ratios (HRs) within the first year post-diagnosis were 2.14 (95% CI: 1.63–2.81) for arrhythmia and 2.71 (95% CI: 1.70–4.33) for HF. After more than 10 years post-diagnosis, the HRs were 1.42 (95% CI: 1.21–1.67) for arrhythmia and 1.28 (95% CI: 1.03–1.59) for HF (Yang et al., 2022). Other studies have suggested that patients with axillary lymph node involvement have a threefold higher risk of cardiotoxicity due to more intensive systemic therapy and increased radiation exposure to sensitive coronary structures (Guo et al., 2025).

Atrial fibrillation (AF), which typically occurs within 2 months after RT completion, is the most common arrhythmia observed in BC patients (Wang et al., 2019). In terms of pathophysiology, AF is conventionally defined as arrhythmia arising from arrhythmogenic ectopic foci located in damaged myocardial tissue within the pulmonary veins (PV) (Lau et al., 2017; Haïssaguerre et al., 1998). Based on these mechanisms, it is likely that radiation-induced PV fibrosis (O'Sullivan and Levin, 2003) contributes to AF as a late sequel of RT (Walls et al., 2024). The maximum dose to PVs (PVd max) appears to be a significant predictor of AF, independent of other risk factors and even after dose adjustment to adjacent cardiac substructures associated with AF (Butler et al., 2024). The incidence of AF is generally higher in BC patients and is largely related to the stage of disease at diagnosis (Guha et al., 2022). Additionally, a cohort study of 560 LC patients demonstrated that the maximum dose delivered to the sinoatrial node was an independent factor associated with atrial fibrillation and overall survival (Kim et al., 2022). RT also causes autonomic dysfunction, most likely secondary to reduced vagal tone. This results in the loss of circadian heart rate variability, persistent tachycardia, and an inappropriate chronotropic response to stress and exercise. Abnormal post-exercise heart rate recovery (HRR) time was observed in 32% of RT patients vs. 9% controls (Groarke et al., 2015). Increased all-cause mortality was also reported over a median follow-up of 3 years in patients with abnormal post-exercise HRR time (Groarke et al., 2015).

Although RT-induced conduction abnormalities are rare, affecting approximately 4%–5% of patients overall, up to 75% of patients receiving mediastinal RT present with ECG abnormalities (Belzile-Dugas and Eisenberg, 2021; Koutroumpakis et al., 2020; Chang et al., 2017b). The conduction system may be directly damaged by radiation in an inflammatory process leading to fibrosis or as a result of fibrosis following myocardial ischaemia (Orzan et al., 1993). RT has been associated with a higher incidence of QT prolongation, ventricular tachycardia, atrioventricular block, fascicular block, and bundle branch block, most commonly right bundle branch block as the right bundle is located anteriorly and lies directly within the radiation field (Chang et al., 2017b; Larsen et al., 1992). A French study involving 3,853 BC patients showed that RT-treated patients had a higher risk of requiring permanent pacemaker implantation (PPMI) compared with the general population, with 28 observed cases versus 13 expected, corresponding to a standardised incidence ratio (SIR) of 2.18 (95% CI: 1.45–3.06]) (Errahmani et al., 2021).

RT can also lead to implantable cardiac device dysfunction (ICSD), which may be either transient or may result in permanent device damage (Zaremba et al., 2016). The recommended safe energy is 6 MV, with dose limits ≤2 Gy for pacemakers and 1 Gy for implantable cardioverter-defibrillators (ICDs) (Sabater et al., 2018). These devices should be regularly monitored both during and after RT, particularly in those who are pacemaker-dependent.

The diagnosis and treatment of radiation-induced arrhythmia require the same diagnostic tools and therapeutic interventions as arrhythmias of other aetiologies, including electrocardiography (ECG), Holter monitoring, antiarrhythmic drugs, and implantation of a pacemaker or ICD.

## 3 Risk factors for RIHD

Multiple factors contribute to RIHD, including prior and ongoing anticancer treatments, as well as modifiable (lifestyle-related) and non-modifiable (sociodemographic characteristics and pre-existing heart disease) patient-related risk factors. It is crucial to identify and modify these factors to reduce the risk of cardiovascular events associated with anticancer therapy while simultaneously preventing premature treatment discontinuation.

### 3.1 RT-associated risk factors

Primary prevention of radiation-induced cardiovascular damage depends on the use of advanced techniques to minimize cardiac exposure. However, it is not always possible to completely avoid cardiac exposure due to the proximity of the heart to the tumour (e.g., during lymph node irradiation in BC). Such approaches can preserve or even enhance RT efficacy while reducing the risk of cancer therapy–related cardiovascular toxicity (CTR-CVT) (Lyon et al., 2022). A key objective of modern RT techniques is to minimize MHD, which is recommended as a more reliable parameter for assessing the risk of RT-induced cardiovascular toxicity, as the administered radiation dose may not accurately represent the actual cardiac exposure (Darby et al., 2013; Maraldo et al., 2015). The BACCARAT study showed that MHD alone is insufficient to accurately predict the individual patient’s dose to LV and coronary arteries, the left anterior descending artery (LAD) in particular. It is therefore necessary to consider dose distribution within these specific cardiac substructures rather than MHD alone for precise assessment of RT-induced cardiotoxicity (Wennstig et al., 2019). For this reason, there are ongoing discussions about the safest radiation dose as well as the most appropriate strategies to minimize the risk of RT-induced CVD (Jacob et al., 2019; Hoppe et al., 2020). The heart is considered a vulnerable organ during RT and its radiation exposure should be as low as possible, as there is no “safe” dose (Darby et al., 2013; van Nimwegen et al., 2016). Strategies to prevent and limit CV complications of RT include modifications of cancer treatment to avoid RT (Fuchs et al., 2019; Radford et al., 2015; Kunkler et al., 2015), as well as modifications of the dose and volume of the RT area, if possible. Modern heart-sparing RT techniques include intensity-modulated photon radiotherapy (IMPT), deep inspiratory breath-hold (DIBH), and respiratory-gated techniques for the treatment of BC (Song et al., 2020), as well as imaging-guided radiotherapy and proton beam irradiation (Dabaja et al., 2018). A population-based study by the Danish Breast Cancer Group showed that patients undergoing CT-guided RT did not have an increased risk of cardiac events in left-sided BC patients compared to right-sided BC patients during the first 10 years post-RT, in contrast to the non-CT-guided RT, where a significantly higher risk of cardiac events was observed in left-sided BC patients (Milo et al., 2021). DIBH is one of the most effective heart-sparing techniques in breast cancer RT. Care should be taken to identify patients who do not benefit from this labour-intensive procedure or who benefit less than an alternative approach: if whole-breast irradiation WBI is necessary - prone positioning, or if lymph node RT is necessary, IMRT may be alternative options (Gaál et al., 2021).

### 3.2 Systemic cancer therapy-related risk factors

Systemic therapy combined with surgery is used both pre- and postoperatively, and both strategies show similar efficacy in primarily resectable tumours (Mauri et al., 2005; Early Breast Cancer Trialists’ Collaborative Group EBCTCG, 2018). However, in recent years, there has been a tendency to use preoperative (neoadjuvant) systemic therapy, as it allows for limiting the scope of surgery in the breast and axilla, and for assessing the individual response to the applied systemic treatment modality (Jassem et al., 2020). Depending on the histopathological diagnosis, systemic treatment includes chemotherapy, hormonal therapy, and/or molecularly targeted therapies. Anthracyclines (doxorubicin and epirubicin), cyclophosphamide, taxanes (doctaxel and paclitaxel), anti-HER2 (trastuzumab, pertuzumab), carboplatin, methotrexate, and tamoxifen are most commonly used. All of these have documented cardiotoxic effects of varying intensity and extent (Bird and Swain, 2008; Schmitz et al., 2012; Cardinale et al., 2015). Although rastuzumab and other HER2-targeted monoclonal antibodies are effective in treating HER2-positive breast cancer, they can cause cardiotoxicity, HF in particular (Copeland-Halperin et al., 2019). Trastuzumab combined with anthracyclines increases the risk of cardiac damage. Studies have shown that 8.7% of trastuzumab-treated patients experience significant decreases in LVEF, which can lead to HF (Jerusalem et al., 2019; Slamon et al., 2011). The addition of pertuzumab to trastuzumab improves cancer outcomes, but may also increase cardiac risk (von Minckwitz et al., 2017). Antibody-drug conjugates such as trastuzumab-emtansine (T-DM1) or trastuzumab-deruxtecan (T-DXd) demonstrate reduced cardiotoxicity compared to trastuzumab alone (Colombo and Rich, 2022).

### 3.3 Patient-related risk factors

Classic cardiovascular risk factors, including hypertension (HT), diabetes mellitus (DM), hyperlipidaemia, a family history of coronary artery disease (CAD), and pre-existing CVD, are associated with an increased risk of RIHD (Lyon et al., 2022; Belzile-Dugas and Eisenberg, 2021). This association was supported by a study in Korean BC patients, which demonstrated that the risk of acute coronary syndromes and cardiac mortality was lower among women without these risk factors (Chang J. S. et al., 2017). This analysis also demonstrated that obesity (a modifiable risk factor) was an independent predictor of cardiac mortality, with 5% increase in the risk of cardiac death for each 1 kg/m^2^ increase in body mass index (BMI) (Chang J. S. et al., 2017). Furthermore, smoking during RT was associated with a threefold higher risk of myocardial infarction (MI) (Hooning et al., 2007). Cardiovascular risk following adjuvant RT has been shown to increase with decreasing levels of physical activity (Chang et al., 2019). Therefore, a baseline CV risk assessment, including blood pressure, lipid profile, fasting glucose, HbA1c, and electrocardiography (ECG), is recommended, along with patient education on maintaining a healthy lifestyle and managing lifestyle-related risk factors (Lyon et al., 2022). Additionally, a 10-year risk of fatal and non-fatal CVD events should be estimated using SCORE2 (age <70 years) or SCORE2-OP (age ≥70 years): age <50 years: low risk <2.5%, moderate risk 2.5% to <7.5%, high risk ≥7.5%; age 50–69 years: low risk <5%; moderate risk 5% to <10%; high risk ≥10%; age ≥70 years: low risk <7.5%, moderate risk 7.5% to <15%, high risk ≥15% (Visseren et al., 2021).

## 4 General principles of RIHD prevention

**FIGURE 1:**
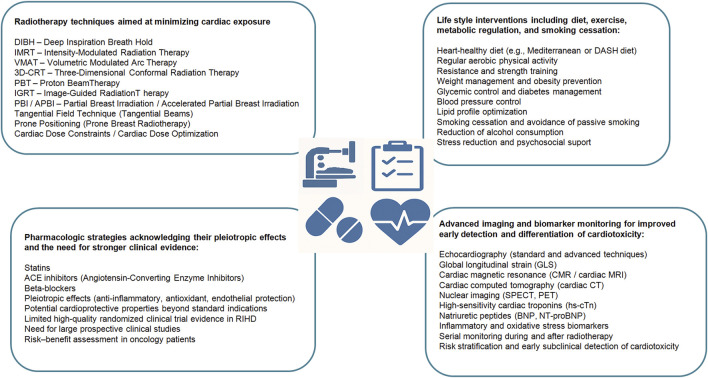
Possibilities of RIHD prevention.

Based on the most recent cardiological (ESC 2022) and oncological (ESMO, ASTRO) guidelines, the following preventive measures have been proposed (Figure 1) (Lyon et al., 2022; Shaitelman et al., 2024; Curigliano et al., 2020; Plana et al., 2014).

### 4.1 Risk assessment before starting RT

CV history and assessment of risk factors (HT, DM, dyslipidaemia, smoking, heart disease), ECG and echocardiography in high-risk patients, biomarker testing (troponin, BNP) – optional.

### 4.2 Optimization of RT

The use of heart-sparing techniques: IMRT, VMAT, proton therapy, DIBH, avoidance of exceeding dose limits: average dose to the heart <5 Gy, minimization of exposure to the left ventricle and coronary arteries.

### 4.3 Modification of CV risk factors

Treatment of HT, DM, and hypercholesterolaemia; smoking cessation; promoting a healthy diet and physical activity.

### 4.4 Pharmacological prophylaxis

Statins in patients with increased cardiac risk; ACE inhibitors or ARBs in patients with HT or left ventricular dysfunction; and beta-blockers in patients with IHD or a history of MI.

### 4.5 Cardiac monitoring after RT

Regular assessment of blood pressure, lipid levels, and glycaemia; echocardiography 6–12 months after completion of RT (in high-risk patients), and then every 2–5 years; biomarkers—to be considered in symptomatic or high-risk patients.

### 4.6 Interdisciplinary care

Collaboration among the oncologist, radiotherapist, and cardiologist, with cardiology consultation in cases of abnormalities or coexisting heart disease.

## 5 Pharmacological prevention of RIHD

There are no proven conservative treatments for preventing RT-induced CV toxicity (Lyon et al., 2022). Although some medications, including statins, ACE inhibitors, and B-blockers, have been reported to contribute to RIHD prevention (Zhang et al., 2015; Huang et al., 2024; O et al., 2009; van der Veen et al., 2015), these are experimental studies. β-blockers, ACE inhibitors, and sodium-glucose cotransporter 2 inhibitors may help improve cardiac dysfunction associated with anticancer therapy, but clinical trials are inconsistent, and therefore individually tailored treatment is needed (Tamburrini et al., 2025).

### 5.1 Statins

Studies in animal and cellular models have shown that statins can reduce oxidative stress, inflammation, and radiation-induced mitochondrial damage by activating adenosine monophosphate-activated protein kinase (AMPK) (Sun et al., 2006). Zhang et al. demonstrated in an experimental rat model that atorvastatin is effective for radiation-induced cardiac fibrosis, particularly with higher doses and longer use (Zhang et al., 2015). A retrospective cohort study in Taiwan involving 1,481 left-sided BC patients who underwent breast-conserving surgery and adjuvant RT demonstrated that statins significantly reduced the risk of major adverse cardiovascular events (MACEs). The 5-year cumulative incidence of MACEs was 12.24% in patients receiving statins, compared with 31.70% of nonusers. The greatest cardioprotective effect was shown for hydrophobic statins, such as rosuvastatin and pravastatin (Huang et al., 2024). These findings suggest a potential role for statins in reducing CV complications in this population and highlight the need for further research to optimize statin therapy. Another meta-analysis demonstrated that statin use was associated with reduced BC mortality for lipophilic statins and decreased all-cause mortality for both lipophilic and hydrophilic statins (Chang et al., 2023; Liu et al., 2017). A meta-analysis of observational studies showed that post-diagnosis statin use was associated with reduced BC mortality, while pre-diagnosis statin use was negatively correlated with both all-cause and cancer-specific mortality (Zhong et al., 2015). On the other hand, a retrospective analysis of 748 patients with locally advanced non-small cell lung cancer (NSCLC) undergoing chest RT found that statin use was inversely associated with survival rates (Atkins et al., 2021). A possible explanation is that patients in the statin group had a higher prevalence of CV risk factors. Therefore, prospective studies are needed to investigate the role of statins in radiation-induced cardiotoxicity and to provide a comprehensive assessment of baseline cardiac risk and cardiac radiation exposure (Jiang et al., 2024). Further research is therefore needed to determine the optimal timing of statin therapy in the context of RT, to identify which patient groups benefit most from such intervention, and to assess the long-term effects of statin use in this population. Currently, the inclusion of statins in the treatment regimen of patients undergoing RT should be considered on an individual basis, taking into account the patient’s overall cardiovascular risk profile.

### 5.2 ACE inhibitors

ACEIs act by inhibiting the renin-angiotensin-aldosterone system (RAAS), leading to a decrease in the production of reactive oxygen species (ROS), reducing oxidative stress and inflammation, increasing the production of nitric oxide (NO), protecting endothelial cells (Dong et al., 2020), and reducing perivascular fibrosis and apoptosis of myocardial cells after cardiac exposure to radiation (Wang et al., 2020). Van der Veen et al. (van der Veen et al., 2015) demonstrated a protective effect of captopril. It improves cardiopulmonary morphology and function by reducing acute cardiac injury. Although preclinical data suggest potential benefits of ACEIs in preventing RIHD, clinical trials confirming their efficacy in this patient population are currently lacking. Therefore, routine use of ACEIs for preventing RIHD in BC patients undergoing RT cannot yet be clearly recommended.

### 5.3 Beta-blockers

Beta-blockers block β1 (and/or β2) adrenergic receptors in the heart, reducing heart rate, which limits myocardial oxygen consumption. They also limit the activation of proinflammatory cytokines, reducing the risk of fibrosis and cardiac dysfunction. They improve endothelial function and coronary blood flow. Some of them (e.g., carvedilol) have antioxidant effects, neutralizing ROS, which protects cardiomyocytes from apoptosis and fibrosis. A retrospective cohort study showed that metoprolol is associated with a reduced risk of CV mortality in BC patients (Tabassum et al., 2024). Another meta-analysis suggested that beta-blockers were associated with longer RFS in patients with early-stage BC, with a more pronounced effect observed in patients with triple-negative breast cancer (TNBC) (Caparica et al., 2021). β-blocker use has also been shown to significantly reduce the risk of death from breast cancer (Childers et al., 2015). However, further research is needed to confirm these findings and understand their mechanism of action.

## 6 Emerging therapeutic strategies with adaptive potential: mesenchymal stem cell–based approaches

In addition to conventional pharmacological cardioprotection, mesenchymal stem cell (MSC)–based therapies have emerged as a promising experimental strategy for mitigating radiation-induced cardiac injury (Wang et al., 2021; Wang et al., 2025). Preclinical studies demonstrate that transplantation of bone marrow-derived MSCs significantly attenuates myocardial damage after cardiac irradiation by enhancing DNA repair pathways, reducing oxidative stress, and improving microvascular integrity (Gao et al., 2017). The therapeutic benefits of MSCs are largely mediated through paracrine mechanisms, including the secretion of vascular endothelial growth factor (VEGF), insulin-like growth factor (IGF), stromal-derived factor-1 (SDF-1), and anti-inflammatory cytokines (Gao et al., 2017; Windmolders et al., 2014). More recently, MSC-derived extracellular vesicles (MSC-EVs) have gained considerable attention as cell-free therapeutic agents capable of modulating apoptosis, inflammation, mitochondrial dysfunction, calcium homeostasis, and fibrotic signaling in irradiated cardiac tissue and cardiac organoid models (Cao et al., 2024; Luo et al., 2021). Despite these encouraging findings, current evidence for MSC-based therapy in RIHD remains limited to experimental and early translational studies (Wang et al., 2021; Wang et al., 2025). Significant challenges—including optimal cell sourcing, delivery strategies, long-term safety, oncologic safety, and reproducibility of therapeutic effects—must be addressed before clinical implementation (Wang et al., 2025; Maria et al., 2016). Nevertheless, MSC-based and MSC-EV–based approaches represent a promising future direction for adaptive and regenerative treatment of radiation-induced cardiac injury (Wang et al., 2021; Kong et al., 2025).

## 7 Conclusion

Advances in BC detection and treatment have led to the need for new research, including more cardioprotective radiotherapy techniques. Due to the complexity of factors influencing cardiovascular risk, both treatment-related and patient-specific risk factors should be considered in treatment planning. These risk factors should be modified at every stage of treatment. Cardiotoxicity significantly compromises the quality of life, even in patients with early-stage BC (Murg et al., 2024), further emphasising the need for early cardiac monitoring and interventions. Further research is needed to assess the impact of radiotherapy on mortality and cardiovascular risk, and to assess the possibility of pharmacological prevention. Raising cardio-oncological awareness and promoting interdisciplinary collaboration are essential to advancing patient care. Initiatives focused on education, improving access to cardio-oncology services, and formal training programmes are essential for addressing any existing shortcomings (Dauccia et al., 2025).
